# A Chemical Proteomics Approach for the Search of Pharmacological Targets of the Antimalarial Clinical Candidate Albitiazolium in *Plasmodium falciparum* Using Photocrosslinking and Click Chemistry

**DOI:** 10.1371/journal.pone.0113918

**Published:** 2014-12-03

**Authors:** Diana Marcela Penarete-Vargas, Anaïs Boisson, Serge Urbach, Hervé Chantelauze, Suzanne Peyrottes, Laurent Fraisse, Henri J. Vial

**Affiliations:** 1 Dynamique des Interactions Membranaires Normales et Pathologiques, CNRS UMR 5235, Université Montpellier II, cc107, Place Eugène Bataillon, 34095 Montpellier Cedex 05, France; 2 Institut de Génomique Fonctionnelle, CNRS UMR 5203, INSERM U661, Université Montpellier I, Université Montpellier II, F-34094 Montpellier, France; 3 Institut des Biomolécules Max Mousseron, CNRS UMR 5247, Université Montpellier II, cc1705, Place Eugène Bataillon, 34095 Montpellier Cedex 05, France; 4 Sanofi, Therapeutic Strategic Unit for Infectious Diseases, 195 route d’Espagne, BP 13669, 31036 Toulouse Cedex, France; Tulane University, United States of America

## Abstract

*Plasmodium falciparum* is responsible for severe malaria which is one of the most prevalent and deadly infectious diseases in the world. The antimalarial therapeutic arsenal is hampered by the onset of resistance to all known pharmacological classes of compounds, so new drugs with novel mechanisms of action are critically needed. Albitiazolium is a clinical antimalarial candidate from a series of choline analogs designed to inhibit plasmodial phospholipid metabolism. Here we developed an original chemical proteomic approach to identify parasite proteins targeted by albitiazolium during their native interaction in living parasites. We designed a bifunctional albitiazolium-derived compound (photoactivable and clickable) to covalently crosslink drug–interacting parasite proteins *in situ* followed by their isolation via click chemistry reactions. Mass spectrometry analysis of drug–interacting proteins and subsequent clustering on gene ontology terms revealed parasite proteins involved in lipid metabolic activities and, interestingly, also in lipid binding, transport, and vesicular transport functions. In accordance with this, the albitiazolium-derivative was localized in the endoplasmic reticulum and trans-Golgi network of *P. falciparum*. Importantly, during competitive assays with albitiazolium, the binding of choline/ethanolamine phosphotransferase (the enzyme involved in the last step of phosphatidylcholine synthesis) was substantially displaced, thus confirming the efficiency of this strategy for searching albitiazolium targets.

## Introduction

*Plasmodium falciparum* (*P. falciparum*) is the etiological agent of severe malaria, a major public health problem which is responsible for almost all malaria deaths and represents an estimated burden of one child dying every minute from malaria in the world [1], [2]. Research on new antimalarial therapies is urgently needed to face the rapid spread of parasite resistance against conventional and recently developed antimalarial drugs [3], [4]. This research should be geared towards discovering new parasite targets and hence new mechanisms of drug action [5].

**Figure 1:**
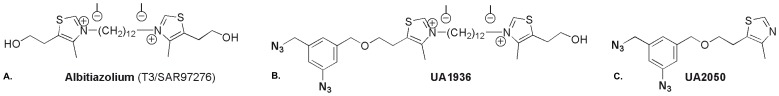
Structure of albitiazolium and photoactivable analogs. (A) The clinical antimalarial candidate albitiazolium, (B) the bifunctional bis-thiazolium derivative UA1936 and (C) the pharmacologically inactive bifunctional derivative UA2050 are depicted. The albitiazolium pharmacophore consists of two cationic thiazolium heads linked by a hydrophobic flexible spacer. The bifunctional compounds UA1936 and UA2050 incorporate a phenyl azido group as photoactivable moiety and a benzy azido group as “clickable” function.

During the *P. falciparum* intraerythrocytic cell cycle, phospholipid synthesis is crucial for sustaining parasite growth and proliferation, which are accompanied by intensive membrane biogenesis [6], [7]. Inhibition of parasite phospholipid biosynthesis has been proposed as a new therapeutic strategy and validated as a pharmacological target for malaria [8], [9]. Phosphatidylcholine is the major phospholipid constituent in *P. falciparum* membranes (40–50%) and is mainly synthesized by the *de novo* Kennedy pathway using choline as precursor [7]. Choline analogs have been designed to inhibit parasite phospholipid metabolism, leading to the development of a new class of antimalarial drugs with an innovative mechanism of action [10], [11]. Among these choline analogs, the bis-thiazolium series have exhibited potent antimalarial activities *in vitro* against *P. falciparum*, with half maximal inhibitory concentrations (IC_50_) ranging from 0.65 to 5 nM, and *in vivo* against *P. vinckei* in mice with half maximal effective doses (ED_50_) ranging from 0.2 to 3.1 mg Kg^−1^ [8], [12], [13]. The T3 lead compound [14], currently named albitiazolium (Figure 1A), has been shown to have appropriate pharmacokinetic and safety parameters in humans and it is being tested in phase II clinical trials by Sanofi, with confirmed antimalarial activity in adult patients.

The mechanism of action of choline analogs is related to their capacity to accumulate specifically and to a high extent inside *P. falciparum* infected erythrocytes [14], [15]. Using a radiolabeled bis-thiazolium derivative, it has been shown that 20% of the drug is localized in the cytoplasm of infected erythrocytes whereas 80% of the accumulated drug is taken up by the parasite. About half of the intraparasitic drug then accumulates in the food vacuole, thus contributing to its antimalarial effect [16]. Recently, we showed that, at pharmacological concentrations, albitiazolium competitively inhibits choline entry into the parasite but also inhibits the three enzymes of the *de novo* pathway of phosphatidylcholine synthesis at higher concentrations [17]. Due to the remarkable *in vitro* antiplasmodial efficacy of albitiazolium, it would be reasonable to hypothesize that albitiazolium could target different molecular activities inside the parasite. The diverse effects on different targets may lead to a synergistic effect relying on diverse biochemical activities (choline transport, membrane biogenesis, food vacule function). This multiple mechanism of action is a substantial advantage by preventing the emergence of drug resistance events.

With the aim of identifying all potential targets of albitiazolium, we designed a chemical proteomics approach for *in situ* capture of proteins targeted by the drug during their native interaction inside living parasites. Choline analogs of the bis-thiazolium series are not metabolized by malaria-infected erythrocytes and they interact in a noncovalent manner with their parasite targets. However, covalent attachment appears crucial to characterize reversible protein-drug interactions using affinity purification based approaches. Consequently, a chemical modification is required in the chemical structure of the drug to enable its irreversible bonding with the targets [18]. In addition, a biochemical tracer needs to be grafted to the drug (e.g. a biotin tag) in order to detect and analyze the protein-drug complexes. These chemical modifications usually impair the intrinsic biological activity of the resulting analogs due to steric problems and/or misdistribution inside the cellular compartments. These drawbacks have led to research on potential drug targets in cellular homogenates instead of whole living cells using drug-immobilized supports. These approaches are however less successful in accurately identifying relevant drug-protein interactions as compared to *in situ* techniques [19]. Our approach overcomes these difficulties through two independent functionalities that have been grafted to the skeleton of the albitiazolium lead compound. We designed and synthesized a bifunctional derivative containing, in addition to the albitiazolium pharmacophore, a phenyl azido photoreactive group [20] to covalently crosslink proteins that interact with the pharmacophore and a small azido group that allows subsequent tagging and purification of interactive proteins following a click chemistry coupling step [21], [22]. The presence of these two functional groups on the same aromatic moiety attached to the drug avoids major steric congestion at the pharmacophore binding site on protein targets, thus permitting the capture of relevant *in situ* drug-protein interactions. Furthermore, through a “clickable” functionality, the tagging of crosslinked complexes offers several possibilities for studying drug-protein interactions, such as whole cell imagery, in-gel detection with fluorescent reporters and affinity purification by specific and irreversible click reactions with biotin or resin-based supports. These bulky tags are inserted once the drug-protein native interaction has taken place [22]. We identified diverse potential albitiazolium targets using this bifunctional compound, which we named UA1936. Remarkably, these proteins are involved in phospholipid metabolism, lipid binding and transport, as well as in vesicular transport functions. To corroborate the specificity of the interaction of these proteins with the bis-thiazolium pharmacophore, we performed competitive experiments in the presence of albitiazolium and obtained a marked reduction in spectral counts for the detection of the choline/ethanolamine phosphotransferase (CEPT) enzyme, which is in line with the proposed mechanism of action of antimalarial bis-thiazolium drugs.

## Results

### Design and synthesis of UA1936, a bifunctional bis-thiazolium compound

A photoactivable and taggable bis-thiazolium compound was synthesized based on the structure of the antimalarial clinical candidate albitiazolium, 3,3′-(dodecan-1,12-diyl)bis[5-(2-hydroxyethyl)-4-methyl-1,3-thiazol-3-ium] dibromide (formerly known as T3 and SAR97276) (Figure 1A). See File S1 for the detailed chemical synthesis. The bifunctional compound was named UA1936 (Figure 1B) and used as bait to search for parasite proteins interacting with choline analogs belonging to the bis-thiazolium series. This compound was designed to be an irreversible ligand by the addition of a phenyl azido photoactivable group, which confers the capacity to covalently capture parasite proteins interacting with bis-thiazolium drugs *in situ*. The UA1936 compound also includes a second azido group in benzylic position, enabling tagging, enrichment and purification of the parasite interactive proteins by click chemistry reactions grafting biotin moieties, fluorescent haptens or alkyne resins.

A second bifunctional derivative harbouring photoactivable and clickable capacities was synthesized as negative control bait. This compound, named UA2050 (Figure 1C), does not have the two cationic heads separated by a hydrophobic flexible 12 carbon-spacer required for antimalarial activity. UA2050 is hence a suitable internal control for detection of the specificity of UA1936 crosslinking due to the absence of the pharmacophore structure of the bis-thiazolium salts.

### The bifunctional UA1936 analog has strong antimalarial activity

**Figure 2:**
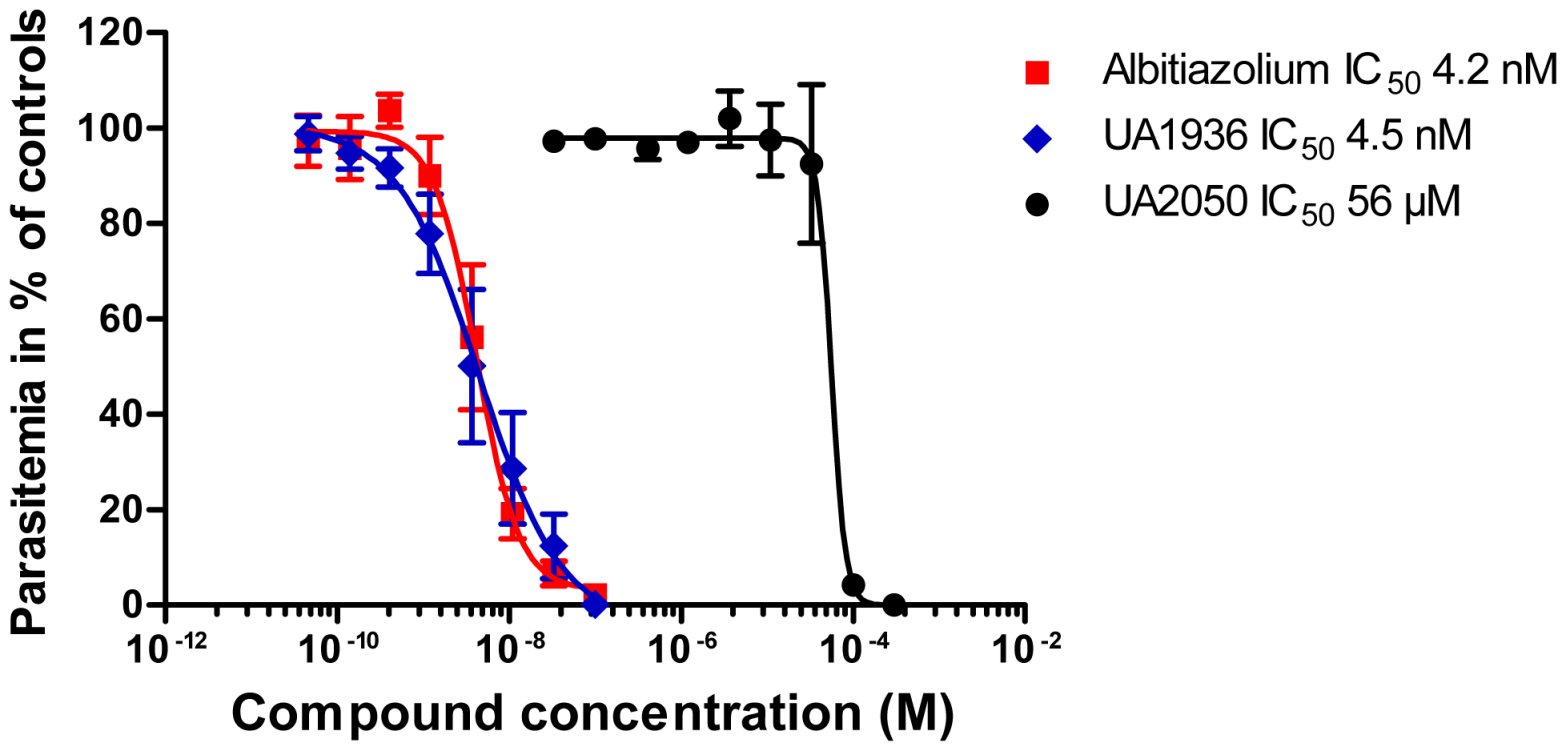
*In vitro* antimalarial activity of albitiazolium and related bifunctional analogs. The compounds were added at various concentrations to the 3D7 *P. falciparum* strain and incubated for 48 h. [3H]hypoxanthine was added at 48 h to monitor parasite viability. Parasitemia is expressed as a percentage of the control cultures without drug. Half maximal inhibitory concentrations (IC_50_) are the concentrations needed to inhibit *P. falciparum* growth by 50%. The results are expressed as means ± SEM of three independent experiments conducted in triplicate. Albitiazolium (□), UA1936 (◊) and UA2050 (○).

The potency of choline analogs of the bis-thiazolium series against the *in vitro* growth of *P. falciparum* has been widely reported elsewhere [12], [14]. Here, the antimalarial activity of the new bifunctional compounds UA1936 and UA2050 were determined in comparison with the clinical candidate albitiazolium. As shown in Figure 2, albitiazolium strongly inhibited *in vitro* plasmodial growth with an IC_50_ value of 4.2 nM. Remarkably, the chemical modifications introduced for the bifunctional UA1936 compound did not alter the intrinsic antimalarial activity of its bis-thiazolium core as its *in vitro* antimalarial activity was very similar to that of the parental albitiazolium drug, with an IC_50_ value in the low nanomolar range (4.5 nM). This indicates that UA1936 is a suitable probe to search for bis-thiazolium targets in *P. falciparum*. On the contrary, the bifunctional UA2050 compound, that lacks the pharmacophore of bis-thiazolium drugs, had substantially impaired antimalarial activity, with an IC_50_ of 56 µM.

### UA1936 is accumulated inside *P. falciparum* and can label parasite-bound proteins

**Figure 3:**
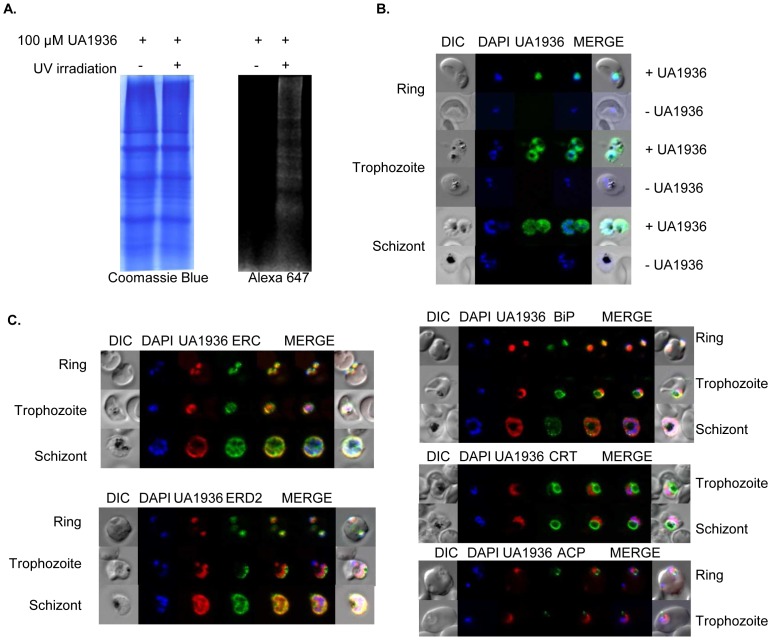
Labeling and detection of UA1936-bound parasite proteins. (A) In-gel fluorescence of UA1936-bound parasite proteins. Saponin isolated parasites were incubated with 100 µM UA1936 and irradiated or not at 254 nm for 2.5 min, parasite proteins were clicked to 10 µM Alexa 647 alkyne. Then 50 µg protein of each sample were separated by SDS-PAGE and imaged in a LiCor OdysseyFc infrared imaging system under an infrared camera. The gel was subsequently stained with Coomassie blue and scanned. (B) and (C). Fluorescence microscopy of UA1936 and its localization in *P. falciparum*-infected red blood cells. *P. falciparum-*infected red blood cells (asynchronous cultures) were cultured with or without 100 µM UA1936 for 1 h and then fixed with 4% paraformaldehyde. In-cell click chemistry was performed with 2.5 µM Alexa 488 alkyne (green) or 2.5 TAMRA alkyne (red) for 30 min. For immunodetection, *P. falciparum*-infected red blood cells were incubated with specific antibodies against the endoplasmic reticulum markers ERC and BiP, against the cis-Golgi marker ERD2, against the food vacuole membrane marker CRT and against the apicoplast marker ACP. Samples were mounted with Vectashield mounting medium with DAPI (blue) and observed under a Zeiss Axioimager epifluorescence microscope.

With the aim of photo-crosslinking and detecting parasite proteins interacting *in situ* with UA1936, saponin-freed living parasites were incubated for 1 h with 100 µM UA1936 under biological culture conditions and irradiated 254 nm for 2.5 min. During this irradiation step, the phenyl azido group present in the UA1936 compound was attached to several parasite proteins that were subsequently labeled with Alexa647 alkyne and revealed by In-gel fluorescence, as shown in Figure 3A. Conversely, when the parasites were incubated with UA1936 but not irradiated, no protein band was detected in the same quantity of proteins loaded on the gel and stained with Coomassie blue (Figure 3A). This shows that the UA1936 compound allowed covalent binding of parasite interacting proteins by UV irradiation in native drug accumulation conditions. Note that, at therapeutic albitiazolium concentrations, the drug increasingly accumulates inside the parasite to reach a concentration of around 1 mM [17]; and hence the level of drug in the vicinity of intracellular target(s) is much higher than the external drug concentration. The incubation of extracellular parasites with UA1936 was limited to 1 h to preserve their viability, so the parasites were incubated with 100 µM UA1936 so as to be in line with a relevant pharmacological concentration.

To further assess the labeling of UA1936-bound parasite proteins and estimate the cellular localization of UA1936 in intact parasites, *P. falciparum*-infected erythrocytes were observed under fluorescence microscopy after 1 h incubation with 100 µM UA1936 followed by in-cell click chemistry reaction with Alexa 488 alkyne. From the early ring to late schizont stages, UA1936 was accumulated and observed specifically inside the parasite cytosol. In mature stages, a strong signal concentration was observed in particular regions inside the parasite (Figure 3B). Attempts to discriminate the cellular compartments of UA1936 accumulation with a battery of specific antibodies revealed partial colocalization of the UA1936 fluorescent signal with the BiP and ERC endoplasmic reticulum markers and with the ERD2 cis-Golgi marker (Figure 3C). No colocalization was found with the CRT food vacuole membrane or the ACP apicoplast markers (Figure 3C). In addition, the UA2050 control compound was also shown to be efficiently accumulated by *P. falciparum*-infected erythrocytes (Figure S1).

### UA1936 interacts with several potential targets in *P. falciparum*

**Figure 4:**
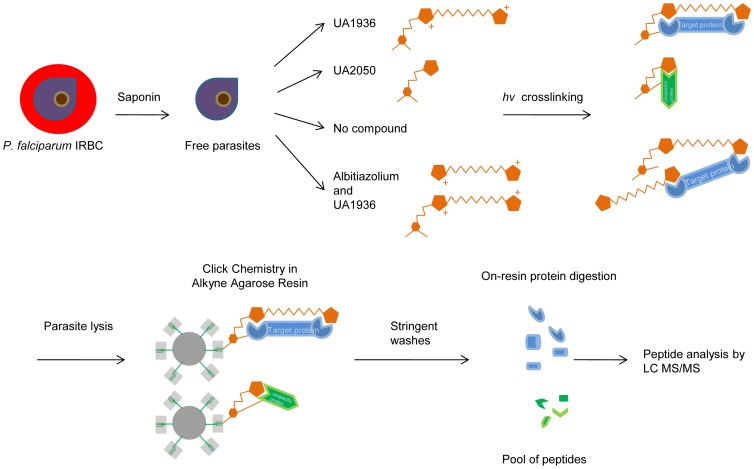
Flow chart for the UA1936-target fishing approach using photocrosslinking and click chemistry. Free parasites were obtained by saponin treatment of *P. falciparum*-infected red blood cells (IRBC) and incubated at 37°C with 100 µM UA1936 or 100 µM UA2050 for 1 h in HEPES-buffered RPMI 1640 medium. Control experiments were also conducted without compound. In competitive experiments, free parasites were first incubated with 100 µM albitiazolium for 30 min and then with 100 µM of UA1936. Parasites were then irradiated at 254 nm for 2.5 min. After centrifugation and wash, parasites were lysed and 10 mg of parasite proteins were used for click chemistry reactions with the alkyne agarose resin. After stringent washes, the resin-bound proteins were digested with trypsin overnight. The peptides were analyzed by mass spectrometry in a LTQ-Orbitrap VELOS mass spectrometer. After spectral data analysis, the identified parasite proteins were clustered based on gene ontology annotations using different bioinformatics packages.

In order to isolate and identify the parasite targets of bis-thiazolium choline analogs, highly stringent conditions were chosen for the purification of UA1936-protein complexes. Knowing that the benzyl azido group present in compound UA1936 allows the irreversible click reaction with resin-based supports, we preferred to immobilize UA1936-protein complexes in an alkyne agarose resin by click chemistry instead of using Biotin/Avidin purification procedures. Indeed, we tested Biotin/Neutravidine purification in preliminary experiments encountering a very low signal-to-noise ratio for the detection of UA1936 partners. On the contrary, with the alkyne agarose resin, it was possible to wash stringently during the purification process, thus considerably reducing the number of irrelevant proteins sticking to the “click resin”, without risk of loss of target proteins. As illustrated in Figure 4, the UA1936-partner proteins irreversibly linked to the alkyne were directly digested on-resin to obtain a pool of peptides for mass spectrometry analysis. The sample of peptides coming from living parasites that had accumulated and crosslinked UA1936 was compared with two control samples of peptides derived from living parasites incubated in the absence of the bifunctional choline analog (no compound) or in the presence of the inactive bifunctional compound UA2050. These experiments were replicated three times and the mass spectrometry results were further analyzed and filtered as described in Materials and Methods.

After the filtering process, eleven proteins were found interacting with UA1936, as shown in Table 1. Two of these parasite proteins were exclusively detected in samples from parasites treated with UA1936, whereas the other nine proteins were detected as highly enriched in samples treated with UA1936 relative to the experimental controls. Importantly, one of the proteins identified exclusively in UA1936-treated parasites was the putative choline/ethanolamine phosphotransferase (PFF1375c-a), the enzyme that accomplishes the final step of the biosynthesis of phosphatidylcholine and phosphatidylethanolamine, the most abundant phospholipid constituents of parasite membranes [6], [7]. The second protein exclusively found in UA1936 treated parasites was a conserved *Plasmodium* protein of unknown function (PFL1815c) and without particular annotation of functional prediction.

**Table 1:** Parasite proteins identified in interaction with the UA1936 bifunctional compound.

			Mean PSM ± SD			Fold enrichment*	
PlasmoDB ID	Previous ID	Name	+ UA1936	-UA1936	+UA2050	+UA1936/−UA1936	+UA1936/+UA2050
PF3D7_ 0628300	PFF1375c-a	choline/ethanolaminephospho transferase, putative (CEPT)	2.3±0.8	0±0	0±0	NC	NC
PF3D7_ 1237500	PFL1815c	conserved *Plasmodium* protein, unknown function	2±0.5	0±0	0±0	NC	NC
PF3D7_ 0904900	PFI0240c	Cu2 -transporting ATPase, putative (CUP)	8±2.5	0.25±0.25	1.50±0.50	32	5.3
PF3D7_ 0112200	PFA0590w	multidrug resistance-associated protein 1 (MRP1)	6.6±1.6	0.25±0.25	1±1	26.6	6.6
PF3D7_ 1412100	PF14_0120	conserved *Plasmodium* protein, unknown function	4.3±1.4	0.25±0.25	2±0	17.3	2.1
PF3D7_ 1242800	PFL2060c	rab specific GDP dissociation inhibitor (rabGDI)	6.6±1.7	0.50±0.25	2±2	13.3	3.3
PF3D7_ 1215900	PFL0765w	conserved *Plasmodium* membrane protein, unknown function (PfSR10)	3.3±1.2	0.25±0.25	0.50±0.50	13	6.6
PF3D7_ 1212500	PFL0620c	glycerol-3-phosphate acyltransferase (Gatp)	2.6±1.2	0.25±0.25	1±1	10.6	2.67
PF3D7_ 0727800	PF07_0115	cation transporting ATPase, putative	2.6±1.2	0.25±0.25	0±0	10	NC
PF3D7_ 1032100	PF10_0314	mRNA-decapping enzyme subunit 1, putative (DCP1)	3.3±1.8	0.50±0.50	0.50±0.50	6.6	6.6
PF3D7_ 1016400	PF10_0160	serine/threonine protein kinase, FIKK family (FIKK10.1)	2.3±0.8	0.50±0.25	0.50±0.50	4.6	4.6

Parasites were incubated in the presence or absence of the UA1936 bifunctional choline analog and in the presence of the UA2050 inactive bifunctional compound. UA1936-interactiing proteins were subsequently photocrosslinked and clicked to an alkyne resin to be identified by mass spectrometry. Means of peptide spectrum matches (PSM) of three independent experiments are indicated for each experimental condition together with the standard deviaton (SD).

*Fold enrichment was calculated by dividing the mean spectral counts of samples incubated with UA1936 by the mean spectral counts of samples incubated either without UA1936 or with UA2050. NC: not calculated when the denominator is zero.

Among the proteins enriched in UA1936 treated parasites, a putative Cu_2_-transporting ATPase, (PFI0240c) was identified with a mean of 8 peptide spectrum matches (PSM) in UA1936 treated parasites, whereas in the control parasites it was identified with a mean PSM of 0.25 and 1.5, respectively, which represents 32- and 5.3-fold enrichment for the detection of this protein in samples treated with UA1936 as compared with the two respective controls. In the same way, the multidrug resistance-associated protein 1 (PFA0590w) was preferentially detected in UA1936 treated parasites, with enrichment ratios of 26.6 and 6.6, respectively. Interestingly the rab specific GDP dissociation inhibitor (PFL2060c) and glycerol-3-phosphate acyltransferase (PFL0620c) were consistently detected with roughly 10- and 3-fold more peptides in parasites treated with UA1936 than in the respective controls. Similarly, a putative cation transporting ATPase (PF07_0115) was found with a 10-fold PSM increase in UA1936 treated samples versus untreated samples and was not detected in samples treated with UA2050. Two conserved *Plasmodium* proteins with unknown function (PF14_0120 and PFL0765w) were also enriched in UA1936 treated samples, as shown in Table 1. Finally, a protein annotated as a putative mRNA-decapping enzyme (PF10_0314) was enriched 6.6-fold and a serine/threonine protein kinase from the FIKK family (PF10_0160) presented a 4.6 enrichment ratio versus both controls.

The exact peptide matches for each protein identified under the different experimental conditions are provided in Table S1. In addition, the differences between the PSM found in UA1936 treated samples and the PSM found in the two controls were analyzed for each protein using a paired t-test, which revealed no significant differences (Table S3).

### Proteins targeted by UA1936 compound are involved in membrane dynamics of *P. falciparum*

**Figure 5:**
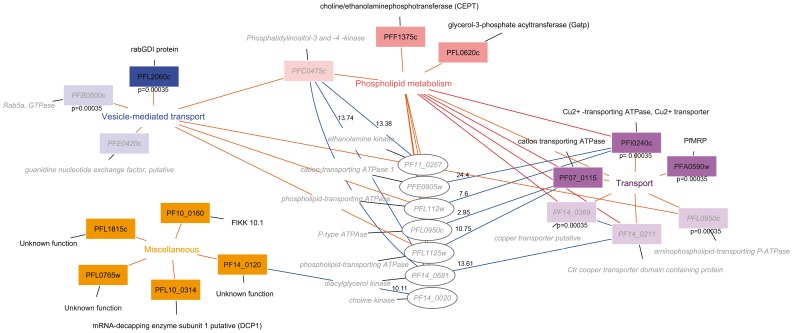
Protein interaction network. Protein interaction networks were built in Cytoscape 2.5.1 using the PlasmoID plugin. Three clusters of UA1936-interacting proteins were found based on the following biological process terms: “phospholipid metabolism” depicted in pink, “vesicle-mediated transport” in blue and “transport” in violet. A fourth cluster of unknown or unrelated functions was created and named “miscellaneous”, as depicted in orange. For each protein (node), the PlasmoDB ID accession number is indicated in a colored rectangle followed by the putative name of the gene. Proteins in dark-colored rectangles were found in the three replicated experiments and proteins in light-colored rectangles were found in two replicates. The proteins depicted in ellipses are interacting proteins identified in the PlamsoMap interactome analysis with their respective likelihood scores. The colors on the edges in the network represent: Codes for (black), Interacts with (blue), and Participates in (orange). The p values for the gene ontology terms found to be significantly enriched are presented for genes having those annotations in the biological process.

A bioinformatic search and interactome analysis of proteins found in interaction with UA1936 was carried out based on existing gene ontology annotations and available protein-protein interaction data [23], [24]. Interestingly, several proteins identified in interaction with the UA1936 compound were involved in membrane-related cellular functions. When the proteins targeted by UA1936 were clustered by gene ontology biological process terms, three categories were found: “phospholipid metabolism”, “vesicle-mediated transport” and “transport”, as shown in Figure 5. Interestingly, when these proteins were clustered by molecular function terms, several of them belonged to “phospholipid binding” and “phospholipid transport” groups. All proteins listed in Table 1 are presented in Figure 5, in addition, a set of proteins that were identified in two out of three of the replicated experiments were included in the bioinformatic analysis and are also shown in pale colored rectangles.

In the group of phospholipid metabolism, two enzymes of the major phospholipid biosynthesis pathway of *P. falciparum* were identified. Remarkably, these enzymes are directly responsible for the glycerophospholipid production of the parasite, i.e. performing the early and final enzymatic steps. Glycerol-3-phosphate acyltransferase (PFL0620c) catalyzes the transfer of one fatty acyl CoA unit to glyceophosphate to form lyso-phosphatidylacid, and choline/ethanolamine phosphotransferase (PFF1375c-a) catalyzes the formation of phosphatdylcholine and phosphatidylethanolamine from CDP-choline and CDP-ethanoloamine, respectively. In addition, a putative phosphatidylinositol 3- and 4-kinase (PFC0475c) that was identified in two out of three of the replicated experiments is also involved in phospholipid metabolism and clustered in this group. Interestingly, plasmoMap showed two interactions for this gene with proteins involved in phospholipid metabolism: PF11_0257 (ethanolamine kinase) and PF14_0681 (putative diacylglycerol kinase), with likelihood scores of 18.38 and 13.74, respectively.

A substantial number of UA1936-interacting proteins were found to be involved in transport processes; importantly, the GO Term Finder tool found significant enrichment of transport ontology terms among potential albitiazolium targets, with a p value of 0.00035, as shown in Figure 5. Among these genes the multidrug resistance-associated protein 1 (PFA0590w), which is involved in the extrusion of glutathione and several antimalarial drugs from chloroquine-resistant parasites [25], a putative Cu^2+^ -transporting ATPase protein containing an E1-E2 domain (PFI0240c) and another cation transporting P-ATPase (PF07_0115). Remarkably, in the PlasmoMAP interactome analysis, the PFI0240c protein appeared to be linked to phospholipid-transporting ATPases, with a high confidence score, as shown in Figure 5. Interestingly, a phospholipid-transporting ATPase protein (PFL0950c) exhibited interaction with UA1936, as well as two putative copper transporter paralogue proteins (PF14_0211 and PF14_0369). These last three proteins are shown in light violet in Figure 5.

A subgroup of UA1937-interacting proteins participates in vesicle-mediated transport processes. In this group, the Rab-specific GDP dissociation inhibitor protein (PFL2060c) was consistently found to be enriched in UA1936-treated parasites. Similarly, two other proteins involved in this biological process were detected in interaction with UA1936 in two experiments, i.e. a guanidine nucleotide exchange factor (PFE0420c) and a Rab GTPase (PFB0500c). The GO Term Finder tool also assigned significant scores for the enrichment of vesicle-mediated transport processes (p = 0.00035).

Three conserved *Plasmodium* proteins with unknown function were clustered in a miscellaneous group. Interestingly among these proteins, PF14_0120, was found to be linked to the choline kinase protein and hence to the phospholipid metabolism with a likelihood score of 10.11. The putative mRNA-decapping enzyme subunit 1 -DCP1 (PF10_0314) was also classified in this group. This putative lipid binding protein contains a Pleckstrin homology domain (PH domain), which mediates the binding of phosphoinositides within biological membranes. A FIKK kinase protein was also clustered in the miscellaneous group. It should be noted that proteins of the FIKK kinase family have been shown to be exported to the host red blood cell [26]. In our approach, parasite proteins exported to the host red blood cell are thought to be lost during saponin treatment and such potential targets might not be captured thereafter. This limitation of our labeling method does not appear to be avoidable in the case of exported soluble proteins. One probable explanation for the capture of the FIKK kinase is that proteins of this family are transported via Maurer’s clefts as membrane bound proteins.

### The interaction of UA1936 compound with choline/ethanolamine phosphotransferase is competitively displaced by albitiazolium

To further define the specificity of the interaction of albitiazolium partners, we assessed whether a reduction in the number of peptide spectrum matches could be obtained during competitive co-incubation of the parasites with both the drug and probe. Table 2 compares the mean PSM for protein identification in the absence and presence of albitiazolium for all potential UA1936 targets identified in the blind fishing approach. Importantly, the highest reduction in spectral counts was observed regarding detection of the choline/ethanolamine phosphotransferase (CEPT) protein, with a 3-fold reduction in the mean peptide spectrum matches detected in parasites when albitiazolium was added. This ratio was calculated by dividing the mean spectral counts of samples incubated with UA1936 by the mean spectral counts of samples incubated with both UA1936 and albitiazolium across two independent replicates. This result indicates that the interaction of UA1936 with choline/ethanolamine phosphotransferase resides in the albitiazolium-pharmacophore part of UA1936 compound. Hence choline/ethanolamine phosphotransferase is a protein that is clearly targeted by bistiazolium drugs.

**Table 2:** Parasite proteins identified in UA1936 treated parasites under competition with albitiazolium.

			Mean PSM ± SD		Fold change reduction*
PlasmoDB ID	Previous ID	Name	+ UA1936	+ UA1936 and Albitiazolium	+ UA1936/+UA1936 and Albitiazolium
PF3D7_ 0628300	PFF1375c-a	choline/ethanolaminephospho transferase, putative (CEPT)	3±1	1±0	**3**
PF3D7_ 1237500	PFL1815c	conserved *Plasmodium* protein, unknown function	1±0	2.3±0.5	0.43
PF3D7_ 0904900	PFI0240c	Cu2 -transporting ATPase, putative (CUP)	15±1	13.5±0.5	1.11
PF3D7_ 0112200	PFA0590w	multidrug resistance-associated protein 1 (MRP1)	14.5±1.5	15±3	0.97
PF3D7_ 1412100	PF14_0120	conserved *Plasmodium* protein, unknown function	13.5±0.5	9±1	1.5
PF3D7_ 1242800	PFL2060c	rab specific GDP dissociation inhibitor (rabGDI)	7.5±2.5	6±0	1.25
PF3D7_ 1215900	PFL0765w	conserved *Plasmodium* membrane protein, unknown function (PfSR10)	6.5±0.5	6.5±1.5	1
PF3D7_ 1212500	PFL0620c	glycerol-3-phosphate acyltransferase (Gatp)	11.5±1.5	8.5±0.5	1.35
PF3D7_ 0727800	PF07_0115	cation transporting ATPase, putative	19±2	16±4	1.19
PF3D7_ 1032100	PF10_0314	mRNA-decapping enzyme subunit 1, putative (DCP1)	1±0	3.5±0.5	0.29
PF3D7_ 1016400	PF10_0160	serine/threonine protein kinase, FIKK family (FIKK10.1)	3.5±1.5	5±1	0.7

Parasites were incubated in the presence of the UA1936 bifunctional compound alone or in co-incubation with albitiazolium. Subsequently UA1936-interactiing proteins were photocrosslinked and clicked to an alkyne resin to be identified by mass spectrometry. Means of peptide spectrum matches (PSM) of two independent experiments are indicated for each experimental condition together with the standard deviaton (SD).

*This ratio was calculated by dividing the mean spectral counts of samples incubated with UA1936 by the mean spectral counts of samples incubated with both UA1936 and albitiazolium across two independent replicates.

Under these competitive conditions, other proteins also presented a discrete reduction in spectral counts: glycerol-3-phosphate acyltransferase was detected with a mean PSM of 8.5 in the presence of albitiazolium versus 11.5 with AU1936 alone (1.35-fold reduction). A conserved *Plasmodium* protein with unknown function, the rab-specific GDP dissociation inhibitor and the cation transporting ATPase showed, respectively, a 1.5-, 1.25- and 1.19-fold PSM reduction in the presence of albitiazolium.

The exact peptide matches for each protein identified under the different experimental conditions are presented in Table S2. In addition, a paired t-test was performed to compare the PSM obtained in UA1936 treated samples versus the PSM of samples incubated with both UA1936 and albitiazolium. The p values showed no statistical differences (Table S3).

## Discussion

We have designed and developed a chemical proteomic approach to capture proteins targeted by antimalarial bis-thiazolium choline analogs during their *in situ* interaction inside live parasites. A bifunctional bis-thiazolium derivative (UA1936) intended to crosslink and tag the parasite targets was synthesized as structural analog of albitiazolium, a current clinical candidate being developed for the treatment of severe malaria.

This bifunctional derivative was shown to retain antimalarial activity and to be taken up by *P. falciparum* under culture conditions, allowing labeling and visualization of the parasite interactive proteins in their cellular context. Interestingly, the compound was predominantly localized in the endoplasmic reticulum and trans-Golgi network within the intracellular parasite (Figure 3). The addition of a phenyl azido photo-reactive group and a benzyl azido “clickable” group did not hamper the antimalarial *in vitro* pharmacological activity of the albitiazolium derivative, with an IC_50_ nearly identical to that of the parental drug (Figure 2), which strongly suggests that the cellular distribution and functions of this derivative remained unchanged regarding the parental compound. Consequently, UA1936 was used as bait to search for parasite proteins targeted by albitiazolium using its two strategic functionalities, i.e. photocrosslinking and click chemistry.

Due to the intrinsic capacity of albitazolium to accumulate in *P. falciparum* infected erythrocytes, the concentration of the drug inside the parasite is several hundredfold superior than the extracellular drug concentration. i.e., millimolar drug concentrations are reached inside the parasite after several hours of incubation with nanomolar therapeutic concentrations of choline analogs in the surrounding medium [15], [16]. In our study, living saponin-freed parasites were incubated with the bis-thiazolium derivative instead of intraerythrocyte parasites since the presence of hemoglobin impedes effective photo crosslinking of the azido-aryl group. As the viability of saponin-freed parasites cannot be preserved for more than 1 h, a 100 µM concentration of the derivative was used to allow the parasites to reach millimolar intracellular drug levels. To assess the feasibility of nonspecific crosslinking of the azido photo-reactive group, a pharmacologically inactive bifunctional compound (UA2050) was also synthesized and tested under the same conditions as the active UA1936 compound. Importantly, the UA2050 control compound was also efficiently accumulated by *P. falciparum*, indicating that the lack of UA2050 antimalarial activity is not related to inefficient accumulation or distribution of the compound inside the parasite. The discrimination of nonspecific crosslinked proteins via photo-reactive compounds was thus achieved by segregation and rejection of the proteins captured in samples treated with the inactive UA2050 compound. In addition, proteins detected in samples treated without any compound were also rejected, reinforcing the specificity of the protein identification.

Mass spectrometry analysis identified diverse potential albitiazolium targets that were consistently detected in three independent experiments. A bioinformatic search and interactome analysis of these different proteins showed that, in addition to their direct involvement in the glycerophospholipid metabolism, these proteins belonged to related biological process like vesicle-mediated transport, phospholipid binding and phospholipid transport. These last sets of proteins could be indirect UA1936 binders through protein-protein interactions because they only presented a slight reduction in spectral counts in albitiazolium-competitive assays. However, their recovery strongly suggests a secondary blockage of membrane dynamics and transport processes, thus contributing to the albitiazolium anti-malarial effect.

For instance, the PH domain-containing protein (PF10_0314) found in interaction with UA1936 has the potential to recognize PIP3 decorated membranes, leading to the recruitment of effector proteins such as guanine nucleotide-exchange factors and Rho GTPases [27]. In line with this, a Rab-specific GDP dissociation inhibitor (PFL2060c) was found in interaction with UA1936. Since Rab cycle proteins are key regulators of membrane fusion and fission processes [28], [29], the interaction of UA1936 with these proteins suggests that UA1936 might concentrate in cellular environments where nascent membranes and budding vesicles form. In agreement with this, we observed high accumulation of the UA1936 compound in the endoplasmic reticulum and cis-Golgi network, i.e. important places for phospholipid biosynthesis and vesicular burgeoning.

Another cluster of UA1936-interacting proteins was involved in transport functions. Two of them were found to be P-ATPases containing E1-E2 domains, with respective putative cation and copper transporter annotations (PF07_0115 and PFI0240c). Proteins containing E1-E2 domains, also known as P-ATPases, can transport different substrates, including ions and phospholipids. Interestingly, a common feature of P-ATPases interacting with UA1936 was their direct or indirect relationship with lipid transport and/or lipid metabolic activities of the parasite *via* protein associations deduced from the interactome data. The potential association of these unexpected proteins with the lipid activities of the parasite requires further investigation by cellular and functional studies. Importantly, a functional role has been established between the P-ATPase-dependent lipid pumping and the endocytic vesicle formation in the yeast plasma membrane [30], as well as in the budding of secretory vesicles in the Glogi network [31]. In this way, P-ATPases with flippase functions can contribute to vesicle-budding by generating membrane bending in the secretory and endocytic pathways [32], [33]. P-ATPases found in interaction with UA1936 might mediate a functional link between the “phospholipid metabolism” and the “vesicle-mediated transport” biological processes targeted by the drug.

The bisthiazolium potential targets identified in our blind fishing approach were further evaluated for their recovery in the presence of the clinical candidate albitiazolium as competitive drug. One key finding of these experiments was that, among the potential targets identified in the proteomic analysis, only choline/ethanolamine phosphotransferase was significantly displaced in the presence of albitiazolium. This finding demonstrates the capacity of our approach in specifically capturing bisthiazolium interacting proteins. Choline/ethanolamine phosphotransferase is crucially involved in the major phospholipid biosynthetic pathways in *P.falciparum*, which is thus in line with the rationale of the albitiazolium drug design. Importantly, the enzymatic activity of this enzyme was recently shown to be inhibited *in vitro* by albitiazolium [17]. Furthermore, this enzyme involved in the last step of phosphatidylcholine and phosphatidylethanolamine biosynthesis in *P. falciparum* was shown to be essential for parasite survival and, in accordance with the cellular localization observed for albitiazolium, is known to be an endoplasmic reticulum resident protein [34], [35]. As further evidence, the choline/ethanolamine phosphotransferase was the only enzyme of the phospholipid metabolism found to be significantly decreased in a proteomic analysis of parasites treated with the T4 bis-thiazolium choline analogue [36], and here we report a 3-fold decrease in spectral counts for its detection in competitive assays with the albitiazolium antimalarial drug. Overall these results indicate that choline/ethanolamine phosphotransferase is a major protein targeted by bistiazolium drugs. Inhibition of this enzyme by these drugs correlates with the decreased production of phosphtidylcholine observed in *P. falciparum* under drug treatment [7], [17]. Noticeably, blockage of choline/ethanolamine phosphotransferase activity does not lead to any other possible pathway for phosphatidylcholine production in *P. falciparum*.

In conclusion, the use of an albitiazolium-derived bifunctional compound revealed a set of *in situ-*interacting proteins in *P. falciparum*. Most of the UA1936-targeted proteins appeared to be associated with the membrane biogenesis and dynamics processes of the intraerythrocytic parasite. These proteins are involved in phospholipid metabolism, but also in vesicular budding and transport functions. Importantly, choline/ethanolamine phosphotransferase displayed a threefold UA1936-label displacement in the presence of albitiazolium, thus supporting the proposed mechanism of action bistiazolium drugs and validating the efficacy of this chemical proteomic approach.

## Materials and Methods

### Synthesis of the bifunctional bis-thiazolium compounds

Details on the synthesis of the photoactivable and clickable bifunctional compounds are described in the supplementary data.

### Parasite culture

The *P. falciparum* 3D7 strain (MRA-102 from MR4) was cultured in human red blood cells at 5% hematocrit in RMPI 1640 medium containing 25 mM HEPES (pH 7.4) and 10% human AB+ serum. Human red blood cells and serum were provided by the French Blood Service “Etablissement français du sang” in accordance with the convention agreement number EFS-PM21/PLER/MTP/CNRSLR01/2013-0049. URL http://www.dondusang.net/rewrite/heading/864/efs/l-efs-en-regions/pyrenees-mediterranee.htm?idRubrique=864. Cultures were maintained at 37°C in a tri-gas mixture of 5% O_2_, 5% CO_2_ and 90% N_2_ [37]. For protein isolation and mass spectrometry studies, large-scale cultures of 8–10 mL packed cell volume of infected erythrocytes at 7 to 10% parasitemia were produced in 636 cm^2^ CellSTACK culture chambers.

### *In vitro* drug sensitivity test

The *in vitro* antimalarial activity of photoactivable alkyl azido compounds was determined against the 3D7 *P. falciparum* strain. Briefly, the *P. falciparum* strain was cultured at 1.5% hematocrit and 0.6% initial parasitemia in microtiter plates in the presence of the compounds for one 48 h cycle. Parasitemia was then assessed by the incorporation of [3H]hypoxanthine in parasite nucleic acids for 12–18 h. Cells were then lysed using an automatic cell harvester and the radioactive nucleic acids were retained on glass fiber paper and counted for radioactivity [38], [39]. The results are expressed as IC_50_ values, which corresponds to the drug concentration leading to 50% inhibition of parasite growth calculated from the plotted parasite growth (expressed as a percentage of the control without drug) versus the log of the compound concentration. Values are means of at least two independent experiments (different cell cultures, different drug dilution stocks), each performed in triplicate.

### UA1936 accumulation in *P. falciparum* infected red blood cells and fluorescence microscopy detection

*P. falciparum* infected red blood cells were cultured in HEPES-buffered RPMI 1640 medium with or without 100 µM UA1936 for 1 h. The infected red blood cells were then washed once with HEPES-buffered RPMI 1640 medium and the cell pellets were fixed with 10 volumes of 4% paraformaldehyde (Electron Microscopy Sciences Cat. # 15710). After washing with 1% BSA in PBS (116 mM NaCl, 8.3 mM Na_2_HPO_4_, 3.2 mM KH_2_PO_4_, pH 7.4), cells were permeabilized with 0.1% Triton X-100 in PBS for 10 min at room temperature and washed again with 1% BSA-PBS. In-cell click chemistry was performed with 2.5 µM Alexa Fluor 488 or TAMRA alkyne for 30 min using the Click-iT cell reaction buffer kit according to the manufacturer’s instructions (Molecular Probes C10269). For immunolabeling, samples were blocked with 3% fetal bovine serum (FBS) in PBS for 1 h at room temperature. Cells were then incubated for 1 h at room temperature with primary antibodies in 3% FBS-PBS as follows: rabbit anti-CRT (anti-chloroquine resistance transporter; MR4 MRA-308) 1∶600, rat anti-ERD2 (anti-ER-retention-defective complementation group 2; MR4 MRA-14) 1∶500, mouse anti-ERC (anti-ER-located calcium binding protein; MRA-87) 1∶1000, rabbit anti-ACP (anti-acyl carrier protein; kindly provided by G. McFadden) 1∶600 and rat anti BiP (anti-immunoglobulin heavy chain binding protein; MR4, J. H. Adams) 1∶1000. After three washes with 1% FBS-PBS, secondary antibodies in 1% FBS-PBS were added for 30 min; anti-rabbit Alexa Fluor 488 (Invitrogen A11012), anti-rat Alexa Fluor 488 (Invitrogen A11007) and anti-mouse Alexa Fluor 488 (Invitrogen A11005). Samples were mounted with Vectashield mounting medium with DAPI and visualized under a Zeiss Axioimager epifluorescence microscope equipped with Apotome. Image acquisition and image analysis were performed on workstations at the Montpellier RIO Imaging facility using Axiovision software.

### UA1936 accumulation in free parasites and photocrosslinking

Free parasites were obtained after saponin treatment of *P. falciparum* infected erythrocytes. Briefly, 10 volumes of 0.02% (wt/vol) saponin in HEPES-buffered RPMI 1640 medium at 4°C were added to 8–10 mL of infected erythrocytes for 3 min and cell suspensions were centrifuged for 10 min at 1,260×g at 4°C, followed by two washes with HEPES-buffered RPMI 1640 medium at 4°C and 10 min centrifugation at 1,260×g. Free parasites were then resuspended in HEPES-buffered RPMI 1640 medium at 1×10^9^ parasites mL^−1^ and incubated at 37°C with 100 µM UA1936 or UA2050 compounds or without drug addition in the media for 1 h in the dark in glass Petri dishes. For competitive assays using albithiazolium, free parasites were first incubated with 100 µM albitiazolium for 30 min and then with 100 µM of UA1936. Parasites were then pelleted and washed once with cold phosphate-buffered saline PBS. Parasites were subsequently resuspended at 1×10^9^ parasites mL^−1^ in cold PBS and placed in glass Petri dishes on ice and irradiated at 254 nm for 2.5 min (Fisher Bioblock Scientific UL215 C 220 V. 50 Hz). After UV irradiation, the parasites were collected by centrifugation at 1 260×g.

### UA1936-bound parasite protein detection by in-gel fluorescence

After UA1936 accumulation and photocrosslinking steps (see above), parasite pellets were lysed at 4°C for 30 min in 1% SDS, 50 mM Tris-HCl, pH 8.0 buffer containing protease inhibitors (Roche 11836 170 001) and sonicated for 1 min by 5 s pulses followed by 5 s incubation on ice. Cell lysates were then centrifuged for 5 min at 16 000×*g* at 4°C and the parasite protein concentrations in the supernatants were measured by the bicinchoninic acid method (Kit BCAssay Uptima FT-40840). 50 µg of parasite proteins was used for each click chemistry reaction with the Click-iT Protein Reaction Buffer Kit (Molecular Probes C10276) according to the manufacturer’s instructions. 10 µM Alexa 647 alkyne (Molecular Probes A10278) was used for cycloaddition reactions under agitation for 30 min at room temperature. After methanol/chloroform precipitation, the proteins were resuspended in electrophoresis sample loading buffer by vortexing and heating for 10 min at 70°C. The samples were then separated by 12% SDS-PAGE. After thoroughly rinsing with water, the gel was scanned at 700 nm in a LiCor OdysseyFc infrared imaging system in order to visualize UA1936-bound parasite proteins.

### UA1936-bound parasite protein isolation using alkyne agarose resin

After UA1936 accumulation in free parasites and photocrosslinking, parasite pellets were resuspended in cold urea lysis buffer, (6 M urea, 200 mM Tris pH 8, 4% CHAPS, 1 M NaCl) supplemented with protease inhibitors (Roche 11836 170 001) on ice for 10 min and then sonicated for 1 min by 5 s pulses followed by 5 s incubation on ice. The lysates were then centrifuged for 10 min at 16 000×*g* at 4°C and parasite protein concentrations in the supernatants were measured by the bicinchoninic acid method (Kit BCAssay Uptima FT-40840). Ten milligrams of parasite proteins (per condition) were used for click chemistry reactions with alkyne agarose resin, according to the manufacturer’s instructions (Click-iT Protein Enrichment Kit, Invitrogen C10416). Briefly, 800 µL of parasite proteins were added to 200 µL of alkyne resin and 1 mL of 2X catalyst solution and the mixtures were incubated at room temperature under rotation overnight. Finally, the reactions were centrifuged for 1 min at 1000×*g* and the supernatant was removed. After reduction and alkylation of the resin-bound proteins with DTT and iodoacetamide, several stringent washes were performed with SDS buffer followed by subsequent washes with 6 M urea buffer and 20% acetonitrile buffer. The agarose resins were then washed with digestion buffer (100 mM Tris, 2 mM CaCl_2_, 10% acetonitrile) and incubated overnight in digestion buffer containing 5 ng µL^−1^ trypsin (Trypsin Gold, V5280 Promega). After 5 min centrifugation at 1000×*g*, supernatants containing the digested peptides were treated with 2% acetonitrile, acidified with trifluoroacetic acid (TFA) and desalted C-18 cartridges. The peptides were then eluted with 50% acetonitrile/0.1% TFA and lyophilized in a SpeedVac concentrator [40]. These experiments were replicated three times.

### Mass spectrometry analysis

The samples were analyzed on line using nanoflow HPLC (Ultimate 3000, Dionex) coupled to a mass spectrometer containing a nanoelectrospray source (LTQ-Orbitrap VELOS, Thermo Fisher Scientific). The peptides were separated in a reverse-phase capillary column (C18, Pepmap, Dionex) over a 0–40% gradient of acetonitrile containing 0.1% formic acid for 150 min at a 300 nL/min flow rate. Spectra were saved using the Xcalibur 2.1 program (Thermo Fisher Scientific) and spectral data were analysed using Proteome Discoverer 1.4 (Thermo Fisher Scientific) and Mascot (Matrix Science) version 2.4 programs. Spectral data were interrogated using CPS human 2013_10 (88266 entries) andPlasmoDB (relase 9.3 including 5538 entries). Spectra were also searched against databases comprising common protein contaminants, using the contaminants_20120713 database (including 247 entries). All databases included carbamidomethylation (C) and oxydation (M) modifications. The mass tolerance was set at 10 ppm for precursor ions and at 0.8 ppm for fragment ions. A maximum of two miscleavages were allowed. Proteins were identified using a target FDR of 0.05. The proteomics experiments were performed using the Functional Proteomics Platform facilities (FPP: http://www.fpp.cnrs.fr/) at the Institut de Génomique Fonctionnelle (Montpellier, France).

### Bioinformatic data analysis

Parasite proteins identified by mass spectrometry were further analyzed and filtered based on two criteria. The first criterion was the reproducibility of the detection of consistent results in the three independent experiments, and the second was the discrimination between nonspecific detected proteins and specific UA1936-bound proteins. All proteins consistently found in samples from parasites incubated without compound were thus considered as background proteins. In the same way, proteins that were consistently found in samples treated with the UA2050 control compound were considered as nonspecific binders not related to the pharmacophore. Finally, proteins that were abundantly detected in UA1936 treated parasites as compared with the two experimental controls were retained for subsequent bioinformatic examination.

Selected proteins were then clustered based on gene ontology annotations using PlasmoDB [41], [42] (release 7.2 and 8.0), Ontology Based pattern Identification [43] UniProt-GOA [44] PlasmoDraft [45] and MPMP (Malaria Parasite Metabolic Pathways) [46] databases. Amino acid sequences of conserved *Plasmodium* proteins of unknown function were also analyzed in OrthoMCL-DB [47]. Clusters and protein interaction networks were built in Cytoscape 2.5.1 [48] using the PlasmoID database [49], which integrates data from different experimental and computational resources, including LaCount DJ [23] and PlasmoMAP [24] datasets. The GO Term Finder tool [50] was used at the web interface http://go.princeton.edu/cgi-bin/GOTermFinder to identify ontology terms significantly enriched among the putative albitiazolium targets. The parameters of the query against a total of 5400 estimated *P. falciparum* gene products and 2307 annotated gene products (from the gene_association.GeneDB_Pfalciparum annotation file) included a p-value cutoff of 0.01 using Bonferroni correction and false discovery rate calculations. This latter value was constantly close to 0%. For the enrichment analysis, an additional set of proteins that were identified in two out of three of the replicated experiments were included based on the high confidence scores assigned for their associations.

### Statistic data analysis

A paired t-test was performed to compare the mean peptide spectral counts obtained in UA1936 treated samples versus the mean PSM in each control condition.

## Supporting Information

Figure S1 **Accumulation of UA2050 compound.** Fluorescence microscopy of UA2050 in *P. falciparum*-infected red blood cells. *P. falciparum-*infected red blood cells (asynchronous cultures) were cultured with 100 µM UA2050 for 1 h and then fixed with 4% paraformaldehyde. In-cell click chemistry was performed with 2.5 µM TAMRA alkyne (red) for 30 min. Samples were mounted with Vectashield mounting medium with DAPI (blue) and observed under a Zeiss Axioimager epifluorescence microscope.
(DOCX)

Table S1 **Peptide sequences of the parasite proteins identified in interaction with the UA1936 bifunctional compound.** The peptide sequences assigned to parasite proteins are indicated for each experimental condition.
(DOCX)

Table S2 **Peptide sequences of the proteins identified in UA1936 treated parasites under competition with albitiazolium.** The peptide sequences assigned to parasite proteins are indicated for each experimental condition.
(DOCX)

Table S3 **P values obtained after t paired test.** Paired comparisons between the peptide spectral matches (PSM) obtained in UA1936 treated samples and the PSM in either controls or competition assay with albitiazolium.
(DOCX)

File S1 **Synthesis of the bifunctional bis-thiazolium compounds.** Detailed chemical synthesis of UA1936 referred as compound 1 and UA2050 referred as compound 2.
(DOCX)
