# Supplementary material for: A Chemical Proteomics Approach for the Search of Pharmacological Targets of the Antimalarial Clinical Candidate Albitiazolium in Plasmodium falciparum Using Photocrosslinking and Click Chemistry
Source: PLoS One. 2014 Dec 3;9(12):e113918. doi: 10.1371/journal.pone.0113918 (PMC4254740; doi:10.1371/journal.pone.0113918)

**Figure S1. Accumulation of UA2050 compound.**

Fluorescence microscopy of UA2050 in *P. falciparum*-infected red blood cells. *P. falciparum-*infected red blood cells (asynchronous cultures) were cultured with 100 µM UA2050 for 1 h and then fixed with 4% paraformaldehyde. In-cell click chemistry was performed with 2.5 µM TAMRA alkyne (red) for 30 min. Samples were mounted with Vectashield mounting medium with DAPI (blue) and observed under a Zeiss Axioimager epifluorescence microscope.


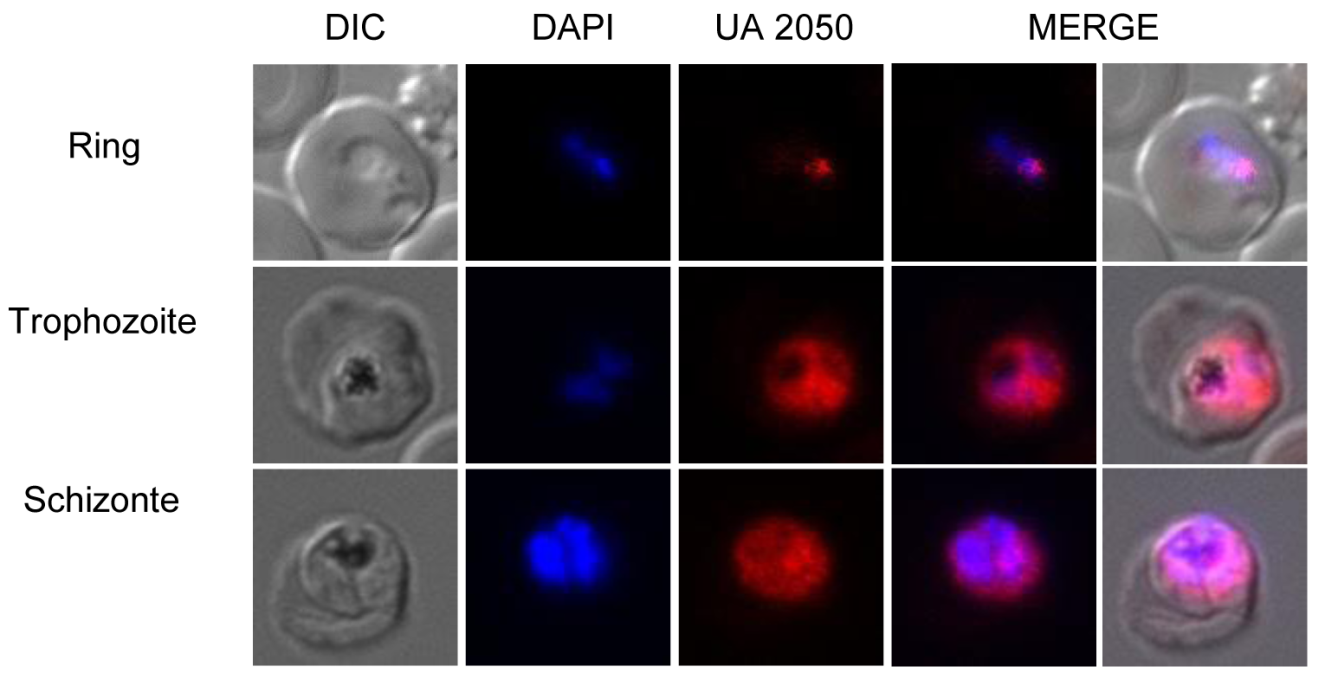

Supplement: Figure S1 — Accumulation of UA2050 compound. Fluorescence microscopy of UA2050 in P. falciparum-infected red blood cells. P. falciparum-infected red blood cells (asynchronous cultures) were cultured with 100 µM UA2050 for 1 h and then fixed with 4% paraformaldehyde. In-cell click chemistry was performed with 2.5 µM TAMRA alkyne (red) for 30 min. Samples were mounted with Vectashield mounting medium with DAPI (blue) and observed under a Zeiss Axioimager epifluorescence microscope. (DOCX) [file pone.0113918.s001.docx]
