# Supplementary material for: A Chemical Proteomics Approach for the Search of Pharmacological Targets of the Antimalarial Clinical Candidate Albitiazolium in Plasmodium falciparum Using Photocrosslinking and Click Chemistry
Source: PLoS One. 2014 Dec 3;9(12):e113918. doi: 10.1371/journal.pone.0113918 (PMC4254740; doi:10.1371/journal.pone.0113918)
Supplement: Table S1 — Peptide sequences of the parasite proteins identified in interaction with the UA1936 bifunctional compound. The peptide sequences assigned to parasite proteins are indicated for each experimental condition. (DOCX) [file pone.0113918.s002.docx]

| **Table S1. Peptide sequences of the parasite proteins identified in interaction with the UA1936 bifunctional compound.**  The peptide sequences assigned to parasite proteins are indicated for each experimental condition. | | | | | | |
| --- | --- | --- | --- | --- | --- | --- |
|  |  |  |  | | |  |
| PlasmoDB ID | Previous ID | Name |  | | |  |
| PF3D7_ 0628300 | PFF1375c-a | choline/ethanolaminephospho transferase, putative (CEPT) | | | | |
|  |  | + UA 1936 | - UA 1937 | | | + UA2050 |
|  |  | VFNTSVGK |  | | |  |
|  |  | TTLKDILPK |  | | |  |
|  |  | GLKGAGVFQR |  | | |  |
|  |  | CTYIGMQNAK |  | | |  |
| PF3D7_ 1237500 | PFL1815c | conserved Plasmodium protein, unknown function | | | |  |
|  |  | + UA 1936 | - UA 1937 | | | + UA2050 |
|  |  | NLFGIPIAR |  | | |  |
|  |  | DSIFGTALLK |  | | |  |
|  |  | SGMNINMLSCDEK |  | | |  |
|  |  | NSNCNIENDHSNNNINEK |  | | |  |
|  |  | TGDIITNDDSLTNNLCIFK |  | | |  |
| PF3D7_ 0904900 | PFI0240c | Cu2 -transporting ATPase, putative (CUP) | | | |  |
|  |  | + UA 1936 | - UA 1937 | | | + UA2050 |
|  |  | HLDINGEILKDDNDISSK | HLDINGEILKDDNDISSK | | | HLDINGEILKDDNDISSK |
|  |  | FEGVEDLK |  | | | FEGVEDLK |
|  |  | EGVEENVVK |  | | |  |
|  |  | AKFEGVEDLK |  | | |  |
|  |  | VVDSYGVIER |  | | |  |
|  |  | GISNVEYDLKK |  | | |  |
|  |  | FSVLNLFNNIK |  | | |  |
|  |  | MFVNTIMNEIK |  | | | MFVNTIMNEIK |
|  |  | LILEGNSFATDNK |  | | |  |
|  |  | HIYSCDCNVHK |  | | |  |
|  |  | SILNSIVSFITDK |  | | |  |
|  |  | NVDAYYDEDKEK |  | | |  |
|  |  | GISCVDESMISGEK |  | | |  |
|  |  | DYLNEDYNNLDK |  | | |  |
|  |  | ESGFNNDLLDLYK |  | | |  |
|  |  | DYLNEDYNNLDKR |  | | |  |
|  |  | SYGDNYQNCNVSEK |  | | |  |
|  |  | GDLYDINYYPNQVEK |  | | |  |
|  |  | NNTLNFVENFSCCNK |  | | |  |
|  |  | EYIYFLYDPELVGIR |  | | |  |
|  |  | YYQDEEGNFNSNIPIK |  | | |  |
| PF3D7_ 0112200 | PFA0590w | multidrug resistance-associated protein 1 (MRP1) | | | |  |
|  |  | + UA 1936 | - UA 1937 | | | + UA2050 |
|  |  | CDNDHILK |  | | |  |
|  |  | DNCSFIISMNK |  | | |  |
|  |  | LFDEVELNHVK |  | | |  |
|  |  | YNEDYNIVVDK |  | | |  |
|  |  | FSSQTEIDEISR |  | | | FSSQTEIDEISR |
|  |  | SSSNMINNPSNFK |  | | |  |
|  |  | FTSIIMPLYVYK |  | | |  |
|  |  | FVENDYIINFIK | FVENDYIINFIK | | | FVENDYIINFIK |
|  |  | LENCSFGLSYDNK |  | | |  |
|  |  | NVSLSSIINSSQDDESK |  | | |  |
|  |  | LVNYNIPFNENYLQK |  | | |  |
|  |  | YTILQSELLNDLSTIEHGDMK | | | |  |
|  |  | YTILQSELLNDLSTIEHGDMK | | | |  |
|  |  | TQSELSHLLEMDD |  | | |  |
| PF3D7_ 1412100 | PF14_0120 | conserved Plasmodium protein, unknown function | | | | |
|  |  | + UA 1936 | - UA 1937 | | | + UA2050 |
|  |  | GMGEYYEFNINK |  | | |  |
|  |  | ELYDYSSSYVNR |  | | |  |
|  |  | VTNNNCFLNSNNK |  | | |  |
|  |  | IPEDITNYITDSFVLLR |  | | | IPEDITNYITDSFVLLR |
|  |  | IDTNDKINNNNSNNNSNNNSK | | | |  |
|  |  | CICLNNLESLEEIEDASLVR | CICLNNLESLEEIEDASLVR | | |  |
|  |  | LNDVIEIIGIYR |  | | | LNDVIEIIGIYR |
| PF3D7_ 1242800 | PFL2060c | rab specific GDP dissociation inhibitor (rabGDI) | | | |  |
|  |  | + UA 1936 | - UA 1937 | | | + UA2050 |
|  |  | FILVGGNLVK | FILVGGNLVK | | | FILVGGNLVK |
|  |  | SSDGEIAYCDK |  | | |  |
|  |  | LNFDDLNTNADGEAPDFN |  | | |  |
|  |  | VPATDMEALVSPLLSLMEK |  | | | VPATDMEALVSPLLSLMEK |
|  |  | SPFIYPLYGLGGIPEGFSR |  | | | SPFIYPLYGLGGIPEGFSR |
|  |  | HWNVDLIPK |  | | |  |
|  |  | MCAINGGTFMLNK |  | | |  |
|  |  | ISDLYVSTSK | ISDLYVSTSK | | |  |
|  |  | NVVDFVFDDDNKVCGIK |  | | |  |
|  |  | VPATDMEALVSPLLSLMEK |  | | | VPATDMEALVSPLLSLMEK |
| PF3D7_ 1215900 | PFL0765w | conserved Plasmodium membrane protein, unknown function (PfSR10) | | | | |
|  |  | + UA 1936 | - UA 1937 | | | + UA2050 |
|  |  | KDMDFK |  | | |  |
|  |  | DTDVYALFLSNCLDSK |  | | |  |
|  |  | GLYIFGENESPYVLLGK |  | | |  |
|  |  | DHDICCYMQEEGIDGYEK |  | | |  |
|  |  | ISHFTEIGDMDEMEDFSNFK |  | | |  |
|  |  |  | LPVYFINDR | | | LPVYFINDR |
|  |  | INTNYILYVLK |  | | |  |
| PF3D7_ 1212500 | PFL0620c | glycerol-3-phosphate acyltransferase (Gatp) | | | |  |
|  |  | + UA 1936 | | - UA 1937 | | + UA2050 |
|  |  | VIENLSYELK | | VIENLSYELK | |  |
|  |  | GKDEFLPILQR | |  | |  |
|  |  | INQTEVYNLVTNSLK | |  | | INQTEVYNLVTNSLK |
|  |  | DLVSTLGPDVSDDFLEQLYR | |  | |  |
|  |  | IESETELLIQEVINIECEDK | |  | |  |
|  |  | NGDTIGIFPEGGSHDR | |  | | NGDTIGIFPEGGSHDR |
| PF3D7_ 0727800 | PF07_0115 | cation transporting ATPase, putative | | | |  |
|  |  | + UA 1936 | - UA 1937 | | | + UA2050 |
|  |  | ENISREEVEK | ENISREEVEK | | |  |
|  |  | FIGDDNVER |  | | |  |
|  |  | VTCNNIESVIK |  | | |  |
|  |  | CSNILYFMNR |  | | |  |
|  |  | ENEEIIGDNEKK |  | | |  |
|  |  | MLQSLDDSLPLIK |  | | |  |
|  |  | VDDVNFNCNDFISK |  | | |  |
|  |  | NIINNNSNNLGGINFR |  | | |  |
| PF3D7_ 1032100 | PF10_0314 | mRNA-decapping enzyme subunit 1, putative (DCP1) | | | | |
|  |  | + UA 1936 | - UA 1937 | | | + UA2050 |
|  |  | NVNEENVNVDQDNEEK | NVNEENVNVDQDNEEK | | |  |
|  |  | EMTTALLNIIK |  | | |  |
|  |  | IINEETNVR |  | | |  |
|  |  | ADIEGFLYIVK |  | | |  |
|  |  | GGEAIMSLLGLSK |  | | |  |
|  |  | NLVETQNENDKR |  | | |  |
|  |  | EILQSDEFIDLLWNK |  | | |  |
|  |  | TGETSHDNINGNNINNNK |  | | | TGETSHDNINGNNINNNK |
| PF3D7_ 1016400 | PF10_0160 | serine/threonine protein kinase, FIKK family (FIKK10.1) | | | |  |
|  |  | + UA 1936 | | | - UA 1937 | + UA2050 |
|  |  | SSDIEQDIDFLR | | | SSDIEQDIDFLR |  |
|  |  | INYINIK | | | INYINIK | INYINIK |
|  |  | VSECDMDIDVFELTR | | |  |  |
|  |  | ICEPLEQLSPITDLDER | | |  |  |
|  |  | EYDIEQNTPDCMFHDLNVFNDILSAR | | | |  |
