# Supplementary material for: A Chemical Proteomics Approach for the Search of Pharmacological Targets of the Antimalarial Clinical Candidate Albitiazolium in Plasmodium falciparum Using Photocrosslinking and Click Chemistry
Source: PLoS One. 2014 Dec 3;9(12):e113918. doi: 10.1371/journal.pone.0113918 (PMC4254740; doi:10.1371/journal.pone.0113918)
Supplement: Table S2 — Peptide sequences of the proteins identified in UA1936 treated parasites under competition with albitiazolium. The peptide sequences assigned to parasite proteins are indicated for each experimental condition. (DOCX) [file pone.0113918.s003.docx]

**Table S2. Peptide sequences of the proteins identified in UA1936 treated parasites under competition with albitiazolium.**

The peptide sequences assigned to parasite proteins are indicated for each experimental condition.

| PlasmoDB ID | Previous ID | Name |  |
| --- | --- | --- | --- |
| PF3D7_ 0628300 | PFF1375c-a | choline/ethanolaminephospho transferase, putative (CEPT) | |
|  |  | + UA 1936 | + UA 1936 and Albitiazolium |
|  |  | SGGNSLLDNLFDAFWNICVK |  |
|  |  | TTLKDILPK |  |
|  |  | IGTTESHVLVITMCILR | IGTTESHVLVITMCILR |
|  |  | GLKGAGVFQR |  |
| PF3D7_ 1237500 | PFL1815c | conserved Plasmodium protein, unknown function | |
|  |  | + UA 1936 | + UA 1936 and Albitiazolium |
|  |  | EINNETNFISNIYNNLDK | EINNETNFISNIYNNLDK |
|  |  |  | NNQTNISEEFINNK |
|  |  |  | NNFIYNYDSGPK |
| PF3D7_ 0904900 | PFI0240c | Cu2 -transporting ATPase, putative (CUP) | |
|  |  | + UA 1936 | + UA 1936 and Albitiazolium |
|  |  | NNTLNFVENFSCCNK | NNTLNFVENFSCCNK |
|  |  | GISCVDESMISGEK | GISCVDESMISGEK |
|  |  | VCMIGDGINDCFALK | VCMIGDGINDCFALK |
|  |  | EYIYFLYDPELVGIR | EYIYFLYDPELVGIR |
|  |  | SILNSIVSFITDK | SILNSIVSFITDK |
|  |  | MFVNTIMNEIK |  |
|  |  | YYQDEEGNFNSNIPIK | YYQDEEGNFNSNIPIK |
|  |  | HLDINGEILKDDNDISSK | HLDINGEILKDDNDISSK |
|  |  | GDLYDINYYPNQVEK | GDLYDINYYPNQVEK |
|  |  | LILEGNSFATDNK | LILEGNSFATDNK |
|  |  | SYGDNYQNCNVSEK | SYGDNYQNCNVSEK |
|  |  | GISNVEYDLKK |  |
|  |  | VYVCTGDNYMNALYISK |  |
|  |  | NVSFYSFTTEK |  |
|  |  | ESGFNNDLLDLYKDDK | ESGFNNDLLDLYKDDK |
|  |  | FSVLNLFNNIK | FSVLNLFNNIK |
|  |  | MFVNTIMNEIK |  |
|  |  | LSLTINNVDDDLKR | LSLTINNVDDDLK |
|  |  |  | DKQNIYICELK |
| PF3D7_ 0112200 | PFA0590w | multidrug resistance-associated protein 1 (MRP1) | |
|  |  | + UA 1936 | + UA 1936 and Albitiazolium |
|  |  | NVSLSSIINSSQDDESK | NVSLSSIINSSQDDESK |
|  |  | LENCSFGLSYDNK | LENCSFGLSYDNK |
|  |  | FSSQTEIDEISR | FSSQTEIDEISR |
|  |  | ENELNVITTQTYK | ENELNVITTQTYK |
|  |  | VINQSNDNTILLTNDCIR | VINQSNDNTILLTNDCIR |
|  |  | SMILFGNEYNPLIYK | SMILFGNEYNPLIYK |
|  |  | LVNYNIPFNENYLQK | LVNYNIPFNENYLQK |
|  |  | YNEDYNIVVDK | YNEDYNIVVDK |
|  |  | TQSELSHLLEMDD | TQSELSHLLEMDD |
|  |  | YTILQSELLNDLSTIEHGDMK | YTILQSELLNDLSTIEHGDMK |
|  |  | YIDEEPSLK | YIDEEPSLK |
|  |  | NFDVPYYASK |  |
|  |  | LFDEVELNHVK | LFDEVELNHVK |
|  |  | RNSLAIIIGNVGSGK |  |
|  |  | LNNYINDHLYYNDIK |  |
|  |  | IFSNLFCK | IFSNLFCK |
|  |  | FVENDYIINFIK | FVENDYIINFIK |
|  |  | IPVYISFLK |  |
|  |  | NSLAIIIGNVGSGK |  |
|  |  | FTSIIMPLYVYK |  |
|  |  | IDNMHHVLKEFK |  |
|  |  |  | DNCSFIISMNK |
|  |  |  | YTILQSELLNDLSTIEHGDMK |
| PF3D7_ 1412100 | PF14_0120 | conserved Plasmodium protein, unknown function | |
|  |  | + UA 1936 | + UA 1936 and Albitiazolium |
|  |  | CICLNNLESLEEIEDASLVR | CICLNNLESLEEIEDASLVR |
|  |  | IPEDITNYITDSFVLLR | IPEDITNYITDSFVLLR |
|  |  | ISLNIFNILYNNEIEK | ISLNIFNILYNNEIEK |
|  |  | ELYDYSSSYVNR |  |
|  |  | NDLSIIISQGPFNDIQK |  |
|  |  | ILAFSDGHNEINR | ILAFSDGHNEINR |
|  |  | LNDVIEIIGIYR | LNDVIEIIGIYR |
|  |  | HNLTDTNNEQKNENIENVK | HNLTDTNNEQKNENIENVK |
|  |  | GIFSSEFYNNINNNYKPNEK | GIFSSEFYNNINNNYKPNEK |
|  |  |  | NENIENVK |
| PF3D7_ 1242800 | PFL2060c | rab specific GDP dissociation inhibitor (rabGDI) | |
|  |  | + UA 1936 | + UA 1936 and Albitiazolium |
|  |  | VPATDMEALVSPLLSLMEK | VPATDMEALVSPLLSLMEK |
|  |  | SPFIYPLYGLGGIPEGFSR | SPFIYPLYGLGGIPEGFSR |
|  |  | FILVGGNLVK | FILVGGNLVK |
|  |  | MCAINGGTFMLNK | MCAINGGTFMLNK |
|  |  | ECILSGLLSHYGK |  |
|  |  | LNFDDLNTNADGEAPDFN |  |
|  |  | VPATDMEALVSPLLSLMEK |  |
|  |  |  | VICDPSYVMHLK |
|  |  |  | VTNYLEWLVVEGSYVYQHQK |
|  |  |  | LNFDDLNTNADGEAPDFN |
|  |  |  | EIEKPLELLGTIEEK |
| PF3D7_ 1215900 | PFL0765w | conserved Plasmodium membrane protein, unknown function (PfSR10) | |
|  |  | + UA 1936 | + UA 1936 and Albitiazolium |
|  |  | DHDICCYMQEEGIDGYEK | DHDICCYMQEEGIDGYEK |
|  |  | DTDVYALFLSNCLDSK | DTDVYALFLSNCLDSK |
|  |  | AAHAIFENVGISTTDNK | AAHAIFENVGISTTDNK |
|  |  | INTNYILYVLK | INTNYILYVLK |
|  |  | YVDEEHMYSLK | YVDEEHMYSLK |
|  |  |  | GLYIFGENESPYVLLGK |
| PF3D7_ 1212500 | PFL0620c | glycerol-3-phosphate acyltransferase (Gatp) | |
|  |  | + UA 1936 | + UA 1936 and Albitiazolium |
|  |  | IESETELLIQEVINIECEDK | IESETELLIQEVINIECEDK |
|  |  | DLVSTLGPDVSDDFLEQLYR | DLVSTLGPDVSDDFLEQLYR |
|  |  | NGDTIGIFPEGGSHDR | NGDTIGIFPEGGSHDR |
|  |  | IKDDEVWMLK | IKDDEVWMLK |
|  |  | LASVIGCISVK | LASVIGCISVK |
|  |  | INQTEVYNLVTNSLK | INQTEVYNLVTNSLK |
|  |  | GKDEFLPILQR | GKDEFLPILQR |
| PF3D7_ 0727800 | PF07_0115 | cation transporting ATPase, putative | |
|  |  | + UA 1936 | + UA 1936 and Albitiazolium |
|  |  | INEINESIINK | INEINESIINK |
|  |  | NIINNNSNNLGGINFR | NIINNNSNNLGGINFR |
|  |  | NDNMNVINNEGVYNK | NDNMNVINNEGVYNK |
|  |  | MNEVISYDLIGEK | MNEVISYDLIGEK |
|  |  | MLQSLDDSLPLIK | MLQSLDDSLPLIK |
|  |  | DDNVNNNFYNFYYK | DDNVNNNFYNFYYK |
|  |  | FFFSSELQR | FFFSSELQR |
|  |  | VTCNNIESVIK | VTCNNIESVIK |
|  |  | ILEQNNYNVK | ILEQNNYNVK |
|  |  | VDDVNFNCNDFISK | VDDVNFNCNDFISK |
|  |  | IGYITIMCGDGTNDMAALK | IGYITIMCGDGTNDMAALK |
|  |  | NQSLENFQIVK | NQSLENFQIVK |
|  |  | KVPEHYDEVLNSLSIK |  |
|  |  | LGEASIASPFTYK | LGEASIASPFTYK |
|  |  | SCFEIPYDIFVHNNVEK |  |
|  |  | NVNEETFVHVK | NVNEETFVHVK |
|  |  | FIGDDNVER |  |
|  |  | IDNDIYFFYK | IDNDIYFFYK |
|  |  | DYEYIEILK | DYEYIEILK |
|  |  | SVINNKSNNNNNK |  |
|  |  | MLQSLDDSLPLIK |  |
|  |  | NDDIKDCIEFLTCLK |  |
| PF3D7_ 1032100 | PF10_0314 | mRNA-decapping enzyme subunit 1, putative (DCP1) | |
|  |  | + UA 1936 | + UA 1936 and Albitiazolium |
|  |  | NVNEENVNVDQDNEEK | NVNEENVNVDQDNEEK |
|  |  |  | EMTTALLNIIK |
|  |  |  | NSALEEIKDYK |
|  |  |  | EILQSDEFIDLLWNK |
|  |  |  | TNVNNINELNNINELNNINK |
| PF3D7_ 1016400 | PF10_0160 | serine/threonine protein kinase, FIKK family (FIKK10.1) | |
|  |  | + UA 1936 | + UA 1936 and Albitiazolium |
|  |  | ICEPLEQLSPITDLDER | ICEPLEQLSPITDLDER |
|  |  | INYINIK | INYINIK |
|  |  | ILFECLNVLR | ILFECLNVLR |
|  |  | VSECDMDIDVFELTR | VSECDMDIDVFELTR |
|  |  |  | NMNGTYLFESCVPTIGK |
|  |  |  |  |
