# Supplementary material for: A Chemical Proteomics Approach for the Search of Pharmacological Targets of the Antimalarial Clinical Candidate Albitiazolium in Plasmodium falciparum Using Photocrosslinking and Click Chemistry
Source: PLoS One. 2014 Dec 3;9(12):e113918. doi: 10.1371/journal.pone.0113918 (PMC4254740; doi:10.1371/journal.pone.0113918)
Supplement: Table S3 — P values obtained after t paired test. Paired comparisons between the peptide spectral matches (PSM) obtained in UA1936 treated samples and the PSM in either controls or competition assay with albitiazolium. (DOCX) [file pone.0113918.s004.docx]

**Table S3. P values obtained after t paired test.**

Paired comparisons between the peptide spectral matches (PSM) obtained in UA1936 treated samples and the PSM in either controls or competition assay with albitiazolium.

| PlasmoDB ID | Previous ID | Name | P value | | |
| --- | --- | --- | --- | --- | --- |
|  |  |  | + UA1936 versus -UA1936 | + UA1936 versus +UA2050 | + UA1936 versus +UA1936 and Albitiazolium |
| PF3D7_ 0628300 | PFF1375c-a | choline/ethanolaminephospho transferase, putative (CEPT) | 0.1181 | 0.1181 | 0.2951 |
| PF3D7_ 1237500 | PFL1815c | conserved *Plasmodium* protein, unknown function | 0.0742 | 0.0742 | 0.2048 |
| PF3D7_ 0904900 | PFI0240c | Cu2 -transporting ATPase, putative (CUP) | 0.0815 | 0.0728 | 0.5 |
| PF3D7_ 0112200 | PFA0590w | multidrug resistance-associated protein 1 (MRP1) | 0.1660 | 0.1515 | 0.7951 |
| PF3D7_ 1412100 | PF14_0120 | conserved *Plasmodium* protein, unknown function | 0.0742 | 0.1885 | 0.2048 |
| PF3D7_ 1242800 | PFL2060c | rab specific GDP dissociation inhibitor (rabGDI) | 0.1022 | 0.1567 | 0.6559 |
| PF3D7_ 1215900 | PFL0765w | conserved *Plasmodium* membrane protein, unknown function (PfSR10) | 0.1217 | 0.0955 | 1 |
| PF3D7_ 1212500 | PFL0620c | glycerol-3-phosphate acyltransferase (Gatp) | 0.1181 | 0.0742 | 0.2048 |
| PF3D7_ 0727800 | PF07_0115 | cation transporting ATPase, putative | 0.1181 | 0.1567 | 0.7048 |
| PF3D7_ 1032100 | PF10_0314 | mRNA-decapping enzyme subunit 1, putative (DCP1) | 0.1567 | 0.1885 | 0.1256 |
| PF3D7_ 1016400 | PF10_0160 | serine/threonine protein kinase, FIKK family (FIKK10.1) | 0.1994 | 0.0742 | 0.6559 |
|  |  |  |  |  |  |
