# Supplementary material for: A Chemical Proteomics Approach for the Search of Pharmacological Targets of the Antimalarial Clinical Candidate Albitiazolium in Plasmodium falciparum Using Photocrosslinking and Click Chemistry
Source: PLoS One. 2014 Dec 3;9(12):e113918. doi: 10.1371/journal.pone.0113918 (PMC4254740; doi:10.1371/journal.pone.0113918)
Supplement: File S1 — Synthesis of the bifunctional bis-thiazolium compounds. Detailed chemical synthesis of UA1936 referred as compound 1 and UA2050 referred as compound 2. (DOCX) [file pone.0113918.s005.docx]

**File S1. Synthesis of the bifunctional bis-thiazolium compounds.**

UA1936 referred as compound 1 and UA2050 referred as compound 2

**3,5-di(hydroxymethyl)aniline (3)**

5-Amino-isophtalic acid dimethyl ether (13.13 g, 62.8 mmol) in dry THF (130 mL) was slowly added into a THF (650 mL) slurry of LiAlH_4_ (6 eq, 14.4 g) at 0°C under vigorous stirring. After stirring at 0°C for 30 min, the mixture was allowed to reflux for 14 hr, and then it was cooled to 0°C. Ethyl acetate (~100 mL) was then slowly added into the grayish mixture under vigorous stirring to quench excess of LiAlH_4_ and *ca.* 75 mL of H_2_O were then added to hydrolyze the alumina salt. A color change from grayish to green was observed. After stirring for another 1 hr, the resulting slurry was filtered, through a pad of silica gel using a coarse frit and washed with several portions of THF (3x500 mL). Solvent removal of the combined filtrates and washings then recrystallization from THF/Hexane to give 3,5-di(hydroxymethyl)aniline (**3**) (5.3 g, 72 %); orange solid; **^1^H** (300 MHz; DMSO) in accordance with the literature.

**1-azido-3,5-di(hydroxymethyl)benzene (4).**

To a solution of 3,5-di(hydroxymethyl)aniline (**2**) (6.7 g, 43.8 mmol) in a 9:1 mixture of acetic acid and water (200 mL) was added NaNO_2_ (1.5 eq, 4.53 g) at 0 °C and the mixture was stirred for 3 min. NaN_3_ (1.5 eq, 4.27 g) at the same temperature and stirring was continued for 15 min. Then saturated aqueous NaHCO_3_ was added solution followed by NaHCO_3_ (powder) until the mixture reached ca. pH 7. The mixture was extracted with EtOAc (×3) and the combined organic layers were washed with brine, dried (MgSO_4_), filtered, and concentrated under reduced pressure. The crude product was recrystallized from EtOAc to give 1-azido-3,5-di(hydroxymethyl)benzene (**4**). (6.66 g, 85 %); orange solid; TLC *Rf* = 0.45 (*n*-hexane/EtOAc = 1/6); **^1^H** (300 MHz; DMSO) δ 4.48 (d, 4H, *J* = 5.8 Hz), 5.27 (t, 2H, *J* = 5.8 Hz), 6.91 (br s, 2H), 7.07 (br s, 1H).

**1-azido-3-bromomethyl-5-(hydroxymethyl)benzene (5)**

To a solution of (**4**) (500 mg, 2.79 mmol) in DMF (6.6 mL) were successively added CBr_4_ (1.5 eq, 1.39 g) and PPh_3_ (1 eq, 805 mg) at 0 °C. After stirring for 2 hr at the same temperature, the mixture was diluted with H_2_O (10 mL) and extracted with CH_2_Cl_2_ (10 mL x2), dried (MgSO_4_), filtered and concentrated under reduce pressure. The crude product was finally purified by silica-gel column chromatography (Petroleum ether then Petroleum ether/EtOAc = 4/1) to give a dibrominated product (110 mg, 13%; brown solid); brown solid; TLC *Rf* = 0.71 (*n*-hexane/EtOAc = 4/1); **^1^H** (300 MHz; CDCl_3_) δ 4.41 (s, 4H), 6.96 (br s, 2H), 7.16 (br s, 1H); **^13^C** (300 MHz, CDCl_3_) δ 31.8 (*C*H_2_Br), 119.5, 126.5 (HC_arom_), 140.2, 141.1 (C_q_) and the desired compound **5** (350 mg, 51%); brown solid; TLC *Rf* = 0.26 (*n*-hexane/EtOAc = 4/1); **^1^H** (300 MHz; CDCl_3_) δ 1.75 (t, 1H, *J* = 6.0 Hz), 4.45 (s, 2H), 4.70 (d, 2H, *J* = 6.0 Hz), 6.97 (br s, 1H), 6.99 (br s, 1H), 7.16 (br s, 1H); **^13^C** (300 MHz, CDCl_3_) δ 33.0 (*C*H_2_Br), 64.2(*C*H_2_OH), 117.9, 119.3, 125.0 (*C*_arom_), 129.7, 141.7, 145.8 (C_q_).

**1-azido-3-azidomethyl-5-(hydroxymethyl)benzene (6)**

To a solution of 1-azido-3-bromomethyl-5-(hydroxymethyl)benzene (**5**) (140 mg, 0.58 mmol) in DMF (2 mL) was added NaN_3_ (2 eq, 75 mg) at room temperature and the mixture was stirred for 2 h. Then water was added and the mixture was extracted twice with CH_2_Cl_2_, dried (MgSO_4_), filtered and concentrated under reduced pressure. The product was purified by silica-gel column chromatography (Petroleum Ether/EtOAc = 4/1) to give the 1-azido-3-azidomethyl-5-(hydroxymethyl)benzene (**6**) (99mg, 84%); pale yellow oil. **^1^H** (300 MHz; CDCl_3_) δ 4.27 (s, 2H, PhC*H*_2_N_3_), 4.65 (s, 2H, PhC*H*_2_O), 6.82 (br s, 1H), 6.97 (br s, 1H), 7.02 (br s, 1H).

**1-azido-3-azidomethyl-5-(4-methylbenzenesulfonyloxymethyl) benzene (7)**

To a solution of Et_3_N (1.4 eq, 1.5 mL), DMAP (0.2 eq, 189 mg) and tosyl chloride (1.4 eq, 2.09 g) in dry DCM (100 mL) was added drop wise 1-azido-3-azidomethyl-5-(hydroxymethyl)benzene (**6**) (1.6 g, 7.8 mmol) at room temperature and stirred for 2 hr. Then water was added and the mixture was extracted twice with CH_2_Cl_2_, dried (MgSO_4_), filtered and concentrated under reduced pressure. The product was purified by silica-gel column chromatography (Petroleum Ether/EtOAc = 9/1) to give the 1-azido-3-azidomethyl-5-(4-methylbenzenesulfonyloxymethyl) benzene (**7**) (2.23g, 80%). **^1^H** (300 MHz; CDCl_3_) δ 2.38 (s, 3H, C*H*_3_), 4.24 (s, 2H, PhC*H*_2_N_3_), 4.96 (s, 2H, PhC*H*_2_O), 6.75 (br s, 1H), 6.84 (br s, 1H), 6.90 (br s, 1H), 7.26 (d, 2H, SCqC*H*_arom_),7.72 (d, 2H, CH_3_CC*H*_arom_).

**1-azido-3-azidomethyl-5-(4-Methyl-5-thiazoleethyloxymethyl) benzene, compound 2 (UA2050)**

1-azido-3-azidomethyl-5-(4-methylbenzenesulfonyloxymethyl)benzene (**7**) (1.33 g, 3.71 mmol) was added drop wise after 30 min to a suspension of NaH (2.3 eq, 205 mg) and 4-methyl-5-thiazoleethanol (1.5 eq, 800 mg) in dry THF (70 mL) and the mixture was stirred for 2 hr. The mixture was diluted with H_2_O, extracted with DCM (x2), dried (MgSO_4_), filtered and evaporated under reduce pressure. The product was purified by silica-gel column chromatography (Petroleum Ether/AcOEt = 9/1 to 3/6) to give the 1-azido-3-azidomethyl-5-(4-Methyl-5-thiazoleethyloxymethyl) benzene (**2**) (1.22 g, 73%); pale yellow oil. **^1^H** (300 MHz; CDCl_3_) δ 2.37 (s, 3H, C*H*_3_), 3.01 (t, 2H, *J* = 6Hz, OCH_2_C*H*_2_), 3.61 (t, 2H, *J* = 6Hz, OC*H*_2_CH_2_), 4.26 (s, 2H, PhC*H*_2_N_3_), 4.46 (s, 2H, PhC*H*_2_O), 6.82 (br s, 1H), 6.91 (br s, 1H), 6.95 (br s, 1H), 8.59 (s, 1H, NC*H*S).

**Compound 8**

To a solution of (**2**) (100 mg, 0.30 mmol) in dry acetonitrile (3 mL) under argon was added 1,2-diiodododecane (5 eq, 640 mg), the mixture was refluxed for 4 days. The product was purified by a silica-gel column chromatography DCM/MeOH (9/1) to give the title compound (**8**) (180 mg, 80%); yellow oil. **^1^H** (300 MHz; CDCl_3_) δ 1.84 (m, 2H, C*H*_2_), 1.99 (m, 2H, C*H*_2_CH_2_N^+^) 2.44 (s, 3H, C*H*_3_), 3.08 (C*H*_2_I) 3.16 (m, 2H, OCH_2_C*H*_2_), 3.70 (m, 2H, CH_2_C*H*_2_O) 4.32 (s, 2H, PhC*H*_2_N_3_), 4.5 (s, 2H, PhC*H*_2_O), 4.61 (m, 2H, C*H*_2_N^+^), 6.85 (br s, 1H), 6.87 (br s, 1H), 7.00 (br s, 1H), 10.85 (s, 1H, N^+^C*H*S).

**Compound 1 (UA1936)**

To a solution of (**8**) (90 mg, 0.119 mmol) in dry acetonitrile (3 mL) was added 4-methyl-5-thiazoleethanol (5eq, 85 mg) under argon, the mixture was refluxed 2 days. The product was purified by a silica-gel column chromatography DCM/MeOH (8/2) to give the desired compound (**1**) (70 mg, 65%); yellow oil; **^1^H** (300 MHz; CD_3_OD) δ 7.13, 6.98, 6.96 (3s, 3H, H_arom_), 4.57 (s, 2H, OC*H*_2_Ph), 4.42 (m, 4H, 2 x N^+^C*H_2_*), 4.37 (s, 2H, N_3_C*H*_2_Ph), 3.77 (m, 4H, 2 x CH_2_C*H_2_*O), 3.20, 3,10 (2t, *J* = 6 Hz, 4H, HOCH_2_C*H_2_*C=C), 2.51 (s, 6H, C*H_3_*C=C), 1.90 (m, 4H, C*H*_2_CH_2_N^+^), 1.37, 1.29 (2br s, 16H, (C*H*_2_)_8_); **^13^C** (300 MHz; CD_3_OD) δ 143.6, 143.3 (N^+^*C_q_)*, 142.1, 139.6 (C_arom_), 137.3, 137.1 (S*C_q_*), 125.1, 119.0, 118.8 (CH_arom_), 73.2 (O*C*H_2_Ph), 61.4 (CH_2_*C*H_2_O), 54.9 (N_3_*C*H_2_Ph), 54.8 (*C*H_2_N^+^), 31.0, 28.5 (C=C*C*H_2_CH_2_OH), 30.4 ((*C*H_2_)_6_), 30.1 (*C*H_2_CH_2_N^+^), 27.3 (*C*H_2_CH_2_CH_2_N^+^) 22.7, (*C*H_2_CH_3_), 12.1, (*C*H_3_C=C); **HPLC** : 8.05 min (97.2%); **MS** (ESI +): 767.3 [M + I]^+^, 320.2 [M^2+^/2]^+^.
